# Co‐Mutation of *ASXL1* and *KRAS* Defines a Novel Ultra‐Adverse‐Risk Subtype of Acute Myeloid Leukemia in a Large‐Scale Cohort

**DOI:** 10.1002/cam4.71715

**Published:** 2026-03-11

**Authors:** Yijing Zhao, Ting Zhao, Feifei Tang, Xiaodong Mo, Yingjun Chang, Xiaosu Zhao, Lanping Xu, Yu Wang, Xiaohui Zhang, Hao Jiang, Qian Jiang, Xiaojun Huang, Yuqian Sun

**Affiliations:** ^1^ Peking University People's Hospital, Peking University Institute of Hematology, Beijing Key Laboratory of Hematopoietic Stem Cell Transplantation Beijing China; ^2^ National Clinical Research Center for Hematologic Disease Beijing Key Laboratory of Hematopoietic Stem Cell Transplantation Beijing China; ^3^ Department of Hematology Peking University People's Hospital Qingdao China

**Keywords:** acute myeloid leukemia, *ASXL1* mutations, *KRAS* mutations, prognosis, relapse

## Abstract

**Background:**

*ASXL1* mutation acute myeloid leukemia represents a clinically aggressive subtype with heterogeneous outcomes. Current evidence remains inconclusive regarding the prognostic relevance of the *KRAS* mutation partner in AML with *ASXL1* mutation. The comprehensive mutational landscape and prognostic implications of co‐occurring driver mutations remain poorly characterized.

**Methods:**

A total of 2788 consecutive AML patient records were reviewed. A comprehensive clinicogenomic analysis was conducted on 451 AML patients with *ASXL1* or *KRAS* mutations from the discovery cohort (*n* = 394) and the independent validation cohort (*n* = 57) to assess the correlation between molecular profiles and clinical outcomes.

**Results:**

The *KRAS* mutation was observed in 22 (9.9%) AML cases with the *ASXL1* mutation. Notably, survival analysis revealed that the *ASXL1* ^*mut*^ */KRAS* ^*mut*^ subtype demonstrated trends toward inferior overall survival (OS) and relapse‐free survival (RFS) relative to *ASXL1* single subgroups. Stratified by mutational status, patients with *ASXL1* ^*mut*^ */KRAS* ^*mut*^ exhibited significantly inferior 2‐year OS rates (30.5% vs. 59.1% vs. 73.9%; *p* < 0.001) and short 2‐year RFS (32.7% vs. 59.4% vs. 69.5%; *p* = 0.002) compared to *ASXL1* ^*mut*^ */KRAS* ^*wt*^ or *ASXL1* ^*wt*^ */KRAS* ^*mut*^ mutation counterparts. This association persisted in the BeatAML invalidation (OS: 0% vs. 43.1% vs. 69.3%; *p* = 0.026). This association persisted in the PSM analysis. This co‐mutation confers an exceptionally poor prognosis comparable to that of *TP53* mutations or complex karyotypes. HSCT showed no significant survival benefit after landmark analysis (OS; *p* = 0.292).

**Conclusions:**

These results demonstrate the independent prognostic value of *ASXL1* ^*mut*^ */KRAS* ^*mut*^ co‐mutation and define a novel ultra‐adverse‐risk subtype of acute myeloid leukemia.

## 1 Introduction

*ASXL1 (the* additional sex comb‐like 1), an epigenetic regulator involved in histone methylation, has emerged as one of the most frequently altered genes in AML, occurring in approximately 10%–20% of AML cases [1]. *ASXL1*‐mutant AML represents a biologically distinct subgroup associated with older age, adverse cytogenetics, and poor survival outcomes characterized by resistance to conventional therapies and a predisposition to relapse [2]. Mutations in *ASXL1* are well‐established as an independent adverse prognostic factor, associated with a higher risk of relapse and shorter overall survival. While the adverse prognostic value of a solitary *ASXL1* mutation is well‐established, its clinical impact is often influenced by its co‐occurring molecular partners. Recent large‐scale genomic studies have revealed that *ASXL1* mutations frequently co‐occur with a variety of other driver mutations, including those in signaling pathway genes such as *KRAS*. However, the precise prognostic relevance of *KRAS* mutations in *ASXL1*‐mutated AML remains inconclusive, and the comprehensive mutational landscape and prognostic implications of these co‐occurring driver mutations are poorly characterized. This study aims to address this knowledge gap by comprehensively analyzing the clinic genomic features and prognostic impact of co‐mutations in a large cohort of *ASXL1*‐mutated AML patients, providing a more refined molecular risk stratification for this clinically aggressive subtype.

We conducted a retrospective cohort study (*n* = 2788) of consecutively AML patients at Peking University People's Hospital (*n* = 2151) as the discovery cohort, and the Beat AML database (*n* = 637) as the validation cohort. We aim to characterize baseline molecular architectures and phenotypic characteristics and determine the association of these variables with prognosis and therapeutic response. By integrating genomic data, clinical information, and clinical outcomes, we aimed to demonstrate the effects of co‐occurring genetic alterations on complete remission (CR) rate, relapse rates, and OS, highlighting the aggressive nature of this molecular phenotype.

## 2 Materials and Methods

### 2.1 Participants

The data of 2151 consecutive patients with AML diagnosed and treated between January 2017 and October 2024 at Peking University People's Hospital (PKUPH) were reviewed. AML was diagnosed based on histology, immunology, cytogenetics, and genetic abnormalities. The inclusion criteria for the study were as follows: (1) age ≥ 16 years; (2) received standard diagnosis and treatment in the medical institution; and (3) had *ASXL1* or *KRAS* mutations. Clinical and follow‐up data were collected retrospectively from electronic medical records, with a final data cutoff date of January 2025. This study was approved by the Ethics Committee of Peking University People's Hospital and adhered to the principles of the Declaration of Helsinki. All the patients signed the appropriate written consent.

### 2.2 Validation Cohort

To independently validate the prognostic significance of *ASXL1/KRAS* co‐mutations, we utilized a publicly available dataset from the BeatAML project [3]. This dataset provides comprehensive genomic and clinical data for a large cohort of AML patients. We extracted data for patients with documented *ASXL1* and *KRAS* mutation status to serve as a validation cohort. The inclusion criteria and statistical analysis for this cohort were consistent with those applied to our primary patient population.

### 2.3 Diagnosis, Monitoring, and Therapy Responses

Diagnosis, monitoring, and treatment methods followed the European LeukemiaNet (ELN) guidelines [4]. Immune phenotyping used multi‐parameter flow cytometry [5]. Cytogenetic analysis was performed using standard G‐banding techniques. Molecular screening for leukemia‐associated fusion genes was conducted for all patients [6]. Clinical data, including blood counts and initial hematologic, cytogenetic, and molecular results, were collected from medical records. Induction regimens, based on those described in prior studies [7, 8], included intensive therapies like HAA (homoharringtonine, cytarabine, aclarubicin) or IA (idarubicin, cytarabine). Patients achieving CR/CRi received high‐dose cytarabine‐based consolidation for 3–4 cycles or less intensive therapy. Non‐responders or relapsed patients were considered for salvage therapies like revised CLAG or FLAG. Patients who were eligible for allo‐hematopoietic stem cell transplantation (HSCT) underwent ≥ 2 consolidation cycles [9, 10, 11]. Donors included HLA‐matched siblings, unrelated donors, or haploidentical related donors [12]. Transplant decisions were based on risk stratification, residual disease, economics, and patient preference [13, 14].

### 2.4 High‐Depth Targeted Regional Sequencing (TRS)

TRS was performed on bone marrow samples from newly diagnosed patients with AML, managed by Kingmed Diagnostics, Guangzhou [15]. The sequencing panel initially covered 175 genes (2018–2020) and then expanded to 290 genes in 2021, focusing on hematologic myeloid neoplasm‐related genes (Table S1). DNA sequencing was conducted using the Illumina NovaSeq6000 platform as per manufacturer protocols. Variant curation followed established cancer variant classification guidelines, with a primary focus on clinically significant (Tier I) and potentially relevant (Tier II) variants.

### 2.5 Statistical Analyses

Overall Survival (OS) was defined as the time from the date of AML diagnosis to death from any cause. Relapse‐Free Survival (RFS) was defined as the time from achieving the first complete remission (CR/CRi) to the date of relapse or death from any cause. The definitions for CR, CRi, and relapse strictly adhered to the ELN recommendations. Descriptive statistics were employed to summarize covariates, with categorical variables presented as counts and percentages and continuous variables as medians and interquartile ranges (IQR). Categorical covariates were analyzed using the Pearson chi‐square test, and continuous covariates were assessed using Student's *t*‐test (for normal distribution) or Mann–Whitney U test (for non‐normal distribution). Multivariable analyses were conducted using Cox regression models to identify covariates associated with OS. Variance inflation factor analysis was performed to evaluate multicollinearity among covariates in the Cox model. OS and response rates were computed using the Kaplan–Meier method and the log‐rank test. Propensity score matching (PSM) was employed to estimate the causal effect of *ASXL1* and *KRAS* co‐mutations on clinical outcomes. Among the participants, covariates, such as age, gender, ELN 2022 risk category, white blood cell count, and whether allo‐HSCT was performed after CR1, were included in the analysis. The matching procedure followed a 1:10 strategy, with each treated participant matched to 10 control participants using nearest‐neighbor matching with a 0.2 caliper. The balance of covariates before and after matching was assessed using standardized mean differences (SMDs) to ensure comparability between groups. A two‐sided *p* < 0.05 was considered statistically significant. Statistical analysis and graphing were performed using SPSS 27.0 (SPSS, Chicago, IL) and R version 4.0.2 (R Core Team, Vienna, Austria).

## 3 Results

### 3.1 Participant Demographics

**FIGURE 1:**
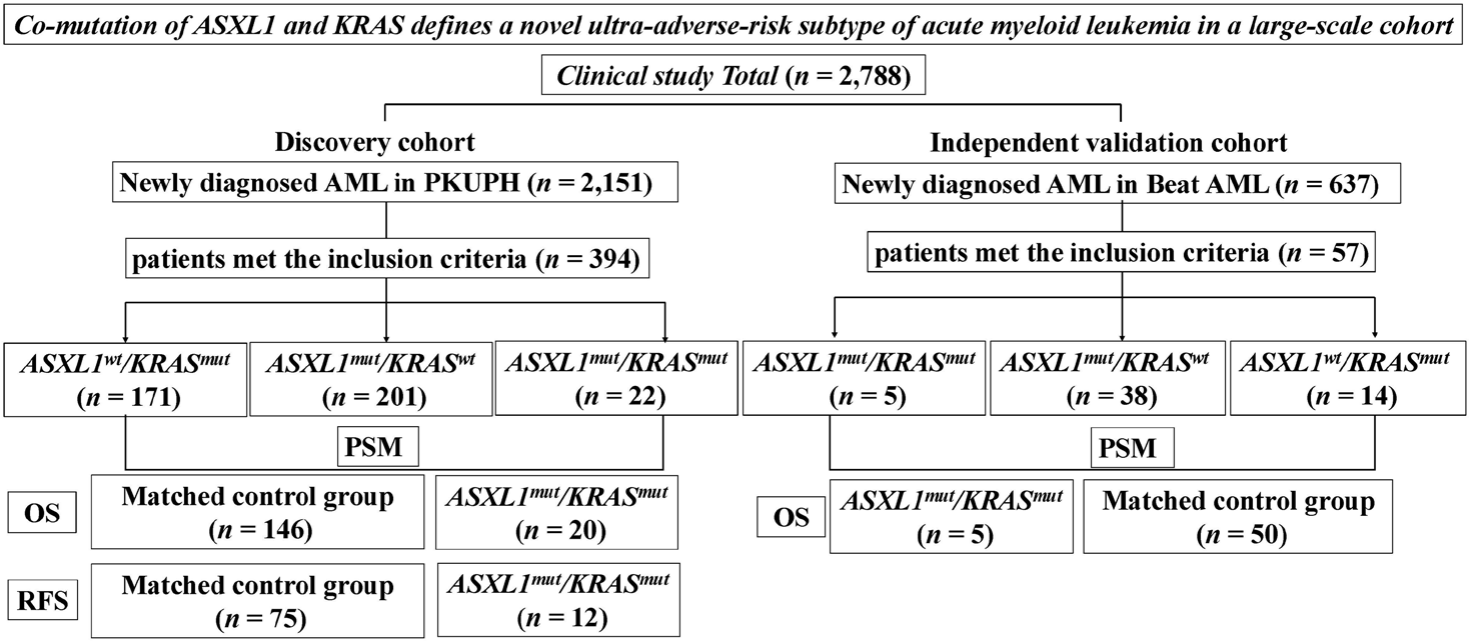
Flowchart of this study.

A total of 2151 consecutive AML patient records were reviewed. Of these, 394 patients met the inclusion criteria of age ≥ 16 years, standard diagnosis and treatment, and the presence of either an *ASXL1* or a *KRAS* mutation. A detailed patient flow diagram illustrating the selection process is provided in Figure 1. The final cohort was composed of 223 patients with an *ASXL1* mutation, 193 with a *KRAS* mutation, and 22 patients with the *ASXL1* ^*mut*^ */KRAS* ^*mut*^ co‐mutation. Clinical characteristics of co‐mutated *ASXL1/KRAS* AML are presented in Table 1. Among the 394 participants deemed eligible for analysis, 137 (34.8%) were female. The median age of the participants was 51 years (IQR, 37–62 years). A total of 296 participants (75.1%) had de novo AML, 84 (23.1%) had antecedent hematologic disorder‐associated AML, and 14 (3.6%) had therapy‐related AML. The activities of daily living score was 95 [85–100]. Risk stratification based on the 2022 ELN (ELN2022) risk classification revealed the following: 124 patients (31.5%) in the favorable risk category, 39 patients (9.9%) in the intermediate risk category, and 231 patients (58.6%) in the adverse risk category (Supplementary Table 2). All 394 patients received induction therapy, distributed as follows: 76 patients (19.3%) received HAA, 107 (27.2%) received IA, 11 (2.8%) received DA, 127 (32.2%) were treated with venetoclax combined with azacitidine, 47 (11.9%) received CAG, and 26 (6.6%) underwent unclassifiable therapy. After the first induction of chemotherapy, 170 (77.78%) patients achieved CR, 47 (13.1%) patients achieved CRi, and 16 (4.4%) patients achieved CRh. Of 394 participants, 134 (34.4%) received transplants after a median of 4 consolidation cycles. Of these, 104 patients (26.4%) underwent allo‐HSCT during CR1. For the 394 patients, the median follow‐up time for survival was 1.26 years. Overall, 103 patients (33.4%) relapsed, and 117 (29.7%) died.

**TABLE 1:** Clinical characteristics of co‐mutated *ASXL1/KRAS* AML.

Number	Level	Overall	*ASXL1* ^*mut*^ *KRAS* ^*wt*^	*ASXL1* ^*mut*^ *KRAS* ^*mut*^	*ASXL1* ^*wt*^ *KRAS* ^*mut*^	*p*
*n*		394	201	22	171	
Sex (%)	Female	137 (34.8)	58 (28.9)	6 (27.3)	73 (42.7)	0.02
	Male	257 (65.2)	143 (71.1)	16 (72.7)	98 (57.3)	
Age (median [IQR])	51.0 [37.0, 62.0]	56.0 [41.0, 64.0]	51.5 [37.5, 66.2]	48.0 [34.0, 56.5]	< 0.01	
*s*AML (%)	De novo AML	296 (75.1)	135 (67.2)	13 (59.1)	148 (86.5)	< 0.01
	AHDAML	84 (21.3)	60 (29.9)	9 (40.9)	15 (8.8)	
	*t*‐AML	14 (3.6)	6 (3.0)	0 (0.0)	8 (4.7)	
WBC (×10^9^/L) (median [IQR])	11.0 [3.5, 35.9]	6.0 [2.3, 29.9]	7.4 [3.8, 22.1]	17.3 [6.6, 43.9]	< 0.01	
HB (g/L) (median [IQR])	81.0 [63.0, 98.2]	79.6 [61.8, 98.0]	82.0 [63.2, 107.5]	82.5 [64.0, 98.0]	0.34	
PLT (×10^9^/L) (median [IQR])	53.0 [27.0, 104.0]	60.0 [28.0, 124.8]	26.0 [18.5, 118.8]	50.0 [27.2, 88.8]	0.18	
ADL (median [IQR])	95.0 [85.0, 100.0]	95.0 [90.0, 100.0]	95.0 [75.0, 100.0]	95.0 [75.0, 100.0]	0.01	
Induction therapy (%)	HAA	76 (19.3)	27 (13.4)	2 (9.1)	47 (27.5)	< 0.01
	IA	107 (27.2)	45 (22.4)	5 (22.7)	57 (33.3)	
	DA	11 (2.8)	4 (2.0)	0 (0.0)	7 (4.1)	
	Ven + AZA	127 (32.2)	76 (37.8)	9 (40.9)	42 (24.6)	
	CAG	47 (11.9)	34 (16.9)	4 (18.2)	9 (5.3)	
	Unclassified	26 (6.6)	15 (7.5)	2 (9.1)	9 (5.3)	
Therapy response						
First therapy (%)	NR/PR	127 (35.3)	71 (38.8)	9 (45.0)	47 (29.9)	0.01
	CR	170 (47.2)	72 (39.3)	6 (30.0)	92 (58.6)	
	CRh	16 (4.4)	8 (4.4)	2 (10.0)	6 (3.8)	
	CRi	47 (13.1)	32 (17.5)	3 (15.0)	12 (7.6)	
Final CR/CRi (%)	No	83 (21.1)	46 (22.9)	10 (45.5)	27 (15.8)	< 0.01
	Yes	311 (78.9)	155 (77.1)	12 (54.5)	144 (84.2)	
Relapse (%)	No	205 (66.6)	95 (62.5)	4 (33.3)	106 (73.6)	0.01
	Yes	103 (33.4)	57 (37.5)	8 (66.7)	38 (26.4)	
HSCT (%)	134 (34.4)	71 (35.7)	8 (38.1)	55 (32.4)	0.75	
HSCT at first CR (%)	104 (26.4)	57 (28.4)	4 (18.2)	43 (25.1)	0.52	
State (%)	Alive	277 (70.3)	129 (64.2)	10 (45.5)	138 (80.7)	< 0.01
	Dead	117 (29.7)	72 (35.8)	12 (54.5)	33 (19.3)	
ELN2022 risk category (%)	Favorable	124 (31.5)	45 (22.4)	0 (0.0)	79 (46.2)	< 0.01
	Intermediate	39 (9.9)	3 (1.5)	0 (0.0)	36 (21.1)	
	Adverse	231 (58.6)	153 (76.1)	22 (100.0)	56 (32.7)	

Abbreviations: ADL, activities of daily living score; AHDAML, antecedent hematologic disorder‐associated acute myeloid leukemia; CLAG, cladribine, cytarabine, and granulocyte colony‐stimulating factor; CR, complete remission; CRH, complete remission with partial hematologic recovery; CRI, complete remission with incomplete blood count recovery; de novo AML, newly diagnosed acute myeloid leukemia without prior hematologic disorders; HAA, homoharringtonine, cytarabine, and aclarubicin; HB, hemoglobin level; HSCT, hematopoietic stem cell transplantation; IA, idarubicin and cytarabine; NR, no response; PLT, platelet count; PR, partial response; sAML, secondary acute myeloid leukemia; t‐AML, therapy‐related acute myeloid leukemia; Ven + AZA, venetoclax combined with azacitidine; WBC, white blood cell.

### 3.2 Mutation Analysis Overview

**FIGURE 2:**
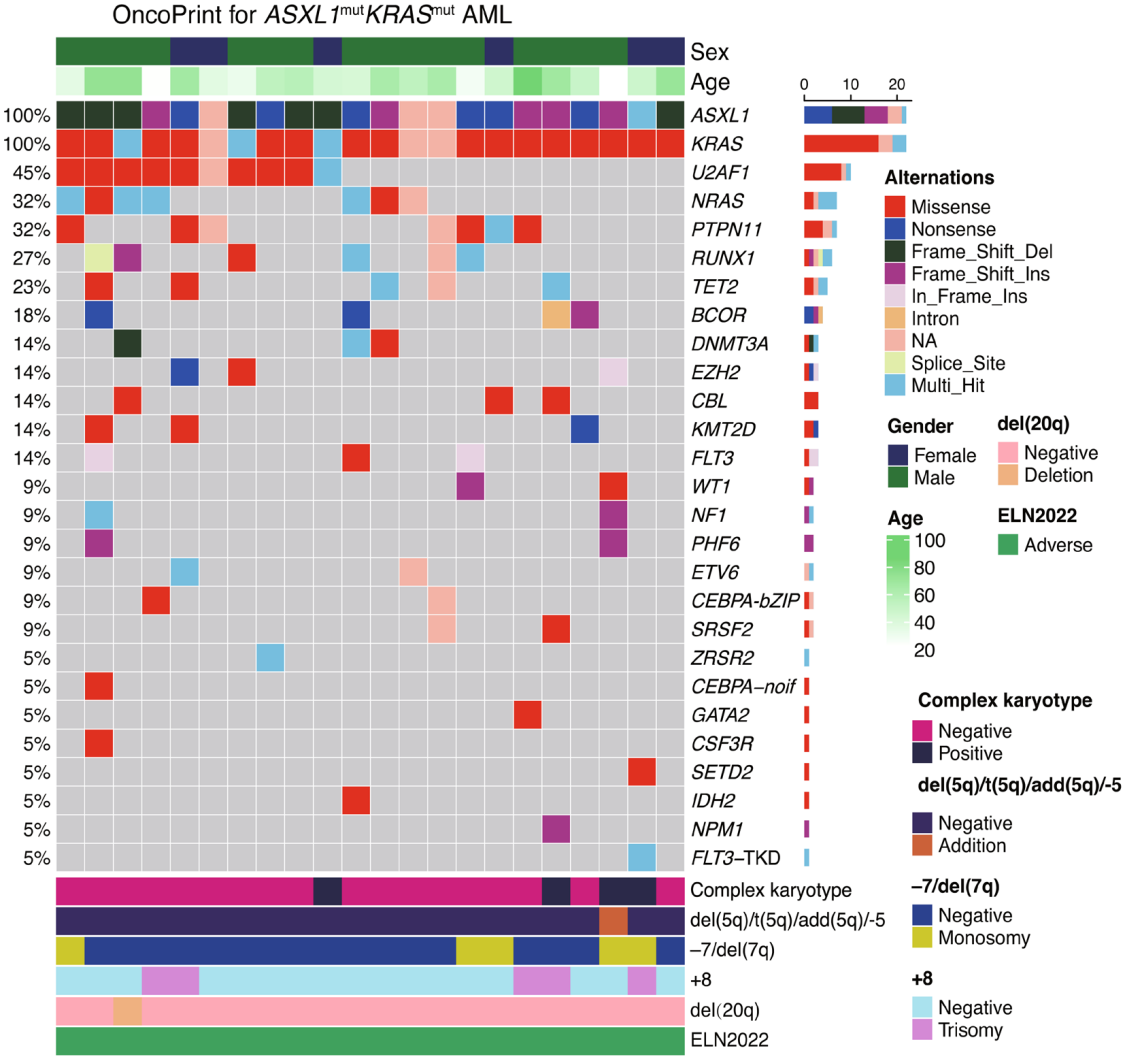
Genetic landscape of co‐mutated *ASXL1* ^mut^/*KRAS* ^mut^ acute myeloid leukemia. *FLT3* (non‐ITD/TKD): Mutations in *FLT3* that are outside the internal tandem duplication (ITD) region and outside the tyrosine kinase domain (TKD). *CEBPA‐bZIP*: In‐frame mutations within the bZIP domain of *CEBPA*. *CEBPA‐noif*: Non–in‐frame mutations within the bZIP domain of *CEBPA*.

The genetic landscape of *ASXL1* ^mut^/*KRAS* ^mut^ AML is shown in Figure 2. The genomic characters of co‐mutated *ASXL1* ^mut^/*KRAS* ^mut^ AML are presented in Supplementary Table 2. Among the somatic mutations, the most frequently altered genes were *U2AF1* (40.9%), *NRAS* (36.4%), *PTPN11* (27.3%), *TET2* (18.2%), followed by *BCOR* (18.2%) and *RUNX1* (18.2%) (Figure 2). Notably, all patients in the *ASXL1* ^mut^/*KRAS* ^mut^ group do not harbor a *TP53* mutation. The *ASXL1* ^wt^ */KRAS* ^mut^ group had a higher mutation rate (14.0%) than that of the other groups. The distinct genomic profiles associated with co‐mutated *ASXL1* ^mut^/*KRAS* ^mut^ AML reveal the heterogeneity of the disease and the potential implications for treatment and prognosis.

### 3.3 Clinical Impact of ASXL1^mut^ /KRAS^mut^ Co‐Mutations in the Discovery Cohort

**FIGURE 3:**
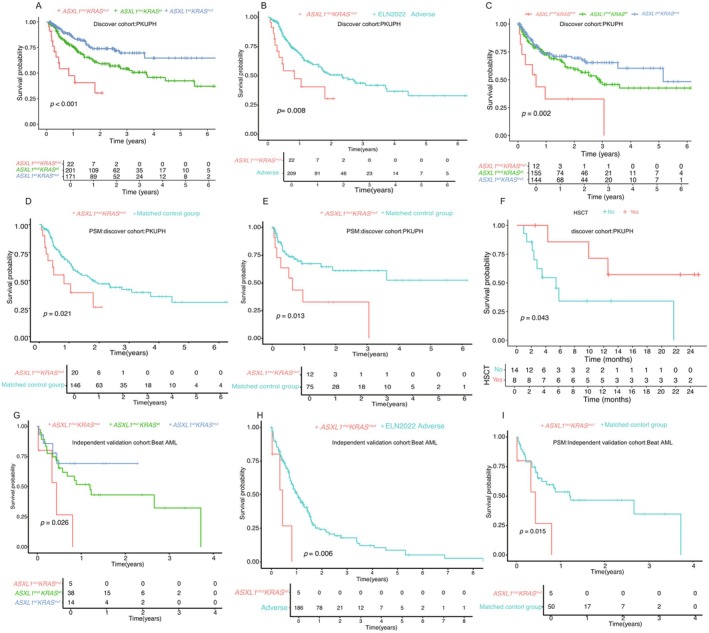
Co‐mutated *ASXL1* ^mut^/*KRAS* ^mut^ AML exhibited poor outcomes. (A) Co‐mutated *ASXL1* ^mut^/*KRAS* ^mut^ AML exhibited poor OS compared with *ASXL1* ^mut^/*KRAS* ^wt^ and *ASXL1* ^wt^/*KRAS* ^mut^ AML in the discovery cohort. (B) Co‐mutated *ASXL1* ^mut^/*KRAS* ^mut^ AML showed poor OS compared with the ELN2022 adverse risk group in the discovery cohort. (C) Co‐mutated *ASXL1* ^mut^/*KRAS* ^mut^ AML demonstrated poor RFS compared with *ASXL1* ^mut^/*KRAS* ^wt^ and *ASXL1* ^wt^/*KRAS* ^mut^ AML in the discovery cohort. (D) Co‐mutated *ASXL1* ^mut^/*KRAS* ^mut^ AML demonstrated poor OS compared with the matched group by PSM in the discovery cohort. (E) Co‐mutated *ASXL1* ^mut^/*KRAS* ^mut^ AML demonstrated poor RFS compared with the matched group by PSM in the discovery cohort. (F) Landmark analysis at 3 months revealed that the survival benefit of HSCT in the *ASXL1* ^mut^/*KRAS* ^mut^ subgroup was not statistically significant. (G) Co‐mutated *ASXL1* ^mut^/*KRAS* ^mut^ AML exhibited poor OS compared with *ASXL1* ^mut^/*KRAS* ^wt^ and *ASXL1* ^wt^/*KRAS* ^mut^ AML in the independent validation cohort. (H) Co‐mutated *ASXL1* ^mut^/*KRAS* ^mut^ AML showed poor OS compared with the ELN2022 adverse risk group in the independent validation cohort. (I) Co‐mutated *ASXL1* ^mut^/*KRAS* ^mut^ AML demonstrated poor OS compared with the matched group by PSM in the discovery cohort. *Abbreviations*: ADV, adverse; AML, acute myeloid leukemia; ELN2022, 2022 European LeukemiaNet; FAV, favorable; HSCT, hematopoietic stem cell transplantation; INT, intermediate; OS, overall survival; RFS, relapse‐free survival.

With respect to clinical outcomes, the *ASXL1* ^mut^/*KRAS* ^mut^ mutation was associated with shorter OS, with a 2‐year OS of 30.5% vs. 59.1% for the *ASXL1* ^mut^ */KRAS* ^wt^ and 73.9% for the *ASXL1* ^wt^ */KRAS* ^mut^ (*p* < 0.001) (Figure 3A, Table S3). In comparison with the ELN2022 adverse risk group, the co‐mutated *ASXL1* ^mut^/*KRAS* ^mut^ AML group demonstrated worse OS with a 2‐year OS of 30.5% vs. 50.7% (*p* = 0.008), further emphasizing the importance of this specific genetic co‐mutation in predicting ultra‐adverse outcomes (Figure 3B, Table S4). Regarding RFS for all CR patients, co‐mutated *ASXL1* ^mut^/*KRAS* ^mut^ AML showed shorter RFS with a 2‐year RFS of 32.7% vs. 59.4% vs. 69.5% (*p* = 0.002) compared with *ASXL1* ^mut^ */KRAS* ^wt^ and *ASXL1* ^wt^ */KRAS* ^mut^ AML, suggesting that these co‐mutations not only affect survival but also the likelihood of relapse (Figure 3C, Table S5).

After PSM, the *ASXL1* ^mut^/*KRAS* ^mut^ mutation was also associated with a shorter OS, with a 2‐year cumulative OS of 26.2% vs. 47% (*p* = 0.021) (Figure 3D). The RFS of the *ASXL1* ^mut^/*KRAS* ^mut^ mutation was also associated with a shorter RFS, with a 2‐year cumulative RFS of 32.7% vs. 61.1% (*p* = 0.013) (Figure 3E). To further evaluate the prognostic standing of the *ASXL1* ^mut^/*KRAS* ^mut^ co‐mutation within the current clinical framework, we compared the outcomes of this subgroup against other established ELN‐2022 adverse‐risk categories, specifically patients with a complex karyotype or *TP53* mutations. In our cohort, the OS and RFS of patients harboring *ASXL1* ^mut^/*KRAS* ^mut^ were strikingly similar to those in the complex karyotype group (*p* = 0.474 for OS; *p* = 0.979 for RFS; Figure S1A,B). Similarly, no significant statistical differences were observed when comparing the *ASXL1* ^mut^/*KRAS* ^mut^ subgroup to patients with *TP53* mutations (*p* = 0.497 for OS; *p* = 0.198 for RFS; Figure S1C,D). These results indicate that the *ASXL1* ^mut^/*KRAS* ^mut^ co‐mutation confers an exceptionally poor prognosis, with clinical outcomes comparable to the most adverse genetic and cytogenetic risk groups defined by the ELN2022 criteria.

Initially, our analysis suggested that HSCT might serve as a significant intervention to alter poor prognostic outcomes in the *ASXL1* ^mut^/*KRAS* ^mut^ subgroup, with patients receiving HSCT showing a longer OS compared to those treated with chemotherapy alone (*p* = 0.043). However, to account for potential immortal time bias, we subsequently performed a 3‐month landmark analysis. In this refined model, the survival advantage previously observed in the HSCT group diminished and reached no statistical significance (*p* = 0.292) (Figure 3F). Specifically, the marginal benefit disappeared after excluding early events occurring before the landmark time. This indicates that the perceived superiority of HSCT in the initial analysis was likely driven by the survival requirement for reaching transplantation rather than a true therapeutic effect. Furthermore, the limited sample size of this subgroup restricts the statistical power to detect smaller differences, suggesting that HSCT may not provide a robust survival advantage for patients harboring both *ASXL1* ^mut^/*KRAS* ^mut^ mutations in this cohort.

### 3.4 Clinical Impact of ASXL1^mut^ /KRAS^mut^ Co‐Mutations in the Independent Validation Cohort

In the validation group, patients with co‐mutated *ASXL1* ^mut^/*KRAS* ^mut^ AML exhibited significantly poorer OS than those with *ASXL1* ^mut^ */KRAS* ^wt^ or *ASXL1* ^wt^ */KRAS* ^mut^ AML, with a 2‐year cumulative OS of 0% vs. 43.1% vs. 69.3% (*p* = 0.026) (Figure 3G, Table S6). In comparison with the ELN2022 adverse risk group, the co‐mutated *ASXL1* ^*mut*^ */KRAS* ^*mut*^ AML group demonstrated worse OS, with a 2‐year cumulative OS of 0% vs. 24.1% (*p* = 0.006), which further emphasizes the importance of this specific genetic co‐mutation in predicting ultra‐adverse outcomes (Figure 3H). After PSM, the *ASXL1* ^mut^/*KRAS* ^mut^ mutation was also associated with a shorter OS, with a 2‐year cumulative OS of 0% vs. 46.4% (*p* = 0.015), in univariable analyses (Figure 3I). Due to the limited CR rate (none of the patients achieved CR), the RFS of the *ASXL1* ^mut^/*KRAS* ^mut^ mutation in the validation group was not performed. These findings independently verified that *ASXL1* ^mut^/*KRAS* ^mut^ mutations are an ultra‐adverse‐risk subtype of acute myeloid leukemia.

### 3.5 Multivariable Analysis

The Cox multivariable regression analysis confirmed that co‐mutated *ASXL1* ^mut^/*KRAS* ^mut^ was an independent poor prognostic factor for OS in patients with AML (HR = 4.88 [2.40, 9.89]; *p* < 0.001). Additionally, age, *SF3B1* mutation, *BCOR* mutation, *TP53* mutation, ELN2022 risk categories, white blood cells, and HSCT were significant independent variables, as shown in Table 2. The Cox multivariable regression analysis for RFS revealed that co‐mutated *ASXL1* ^mut^/*KRAS* ^mut^ was an independent poor prognostic factor (HR = 2.859 [1.320–6.192]; *p* = 0.008). As shown in Table 2, *KMT2D* mutation, *SF3B1* mutation, ELN2022 risk categories, *ETV6* mutation, complex karyotype, and HSCT were also significant independent variables. The statistical analysis was adjusted for various clinical and biological covariates, reinforcing the conclusion that *ASXL1* ^mut^/*KRAS* ^mut^ co‐mutation has a substantial impact on survival.

**TABLE 2:** The multivariate analysis of OS and RFS in patients with *ASXL1* ^*mut*^ *or KRAS* ^*mut*^.

RFS			OS		
Variable	HR (95% CI)	*p*	Variable	HR (95% CI)	*p*
*KMT2D*	5.912 (1.398–24.996)	0.016	FAV	Ref	
*SF3B1*	4.956 (1.172–20.963)	0.03	INT	12.129 (4.921–29.893)	< 0.001
FAV	Ref		ADV	11.29 (5.501–23.172)	< 0.001
INT	4.507 (1.987–10.221)	< 0.001	*SF3B1*	6.102 (1.78–20.921)	0.004
ADV	4.167 (2.359–7.362)	< 0.001	*ASXL1* ^*mut*^ *KRAS* ^*mut*^	4.875 (2.402–9.892)	< 0.001
*ETV6*	3.15 (1.124–8.829)	0.029	*TP53*	2.368 (1.101–5.093)	0.027
t(9;11) (p21.3;q23.3)	2.885 (0.98–8.496)	0.054	Age	1.017 (1–1.035)	0.056
*ASXL1* ^*mut*^ *KRAS* ^*mut*^	2.859 (1.32–6.192)	0.008	WBC (×10^9^/L)	1.006 (1.001–1.012)	0.026
*EZH2*	2.598 (0.904–7.469)	0.076	*BCOR*	0.282 (0.119–0.668)	0.004
Complex karyotype	2.161 (1.048–4.456)	0.037	HSCT	0.122 (0.068–0.218)	< 0.001
*SRSF2*	1.812 (0.99–3.318)	0.054			
HSCT	0.286 (0.178–0.46)	< 0.001			

Abbreviations: ADV, risk category (ELN2022) adverse; AML, acute myeloid leukemia; FAV, risk category (ELN2022) favorable; HSCT, hematopoietic stem cell transplantation; INT, risk category (ELN2022) intermediate; OS, overall survival; RFS, relapse‐free survival; WBC, white blood cells.

## 4 Discussion

The findings of this study provide important insights into the prognostic implications of *ASXL1* ^mut^/*KRAS* ^mut^ co‐mutations in AML, highlighting their role as a distinct molecular subset with aggressive clinical behavior. Patients with *ASXL1* ^mut^/*KRAS* ^mut^ exhibited significantly lower CR/CRi rates, higher relapse rates, shorter RFS, and shorter OS than those with single mutations in *ASXL1* or *KRAS*, aligning with prior evidence of the adverse prognostic impact of these mutations independently. Importantly, we found preliminary evidence that suggests that HSCT may not offer a survival benefit, nor mitigate the adverse prognostic impact of *ASXL1/KRAS* co‐mutations. These observations highlight the importance of identifying and stratifying patients with AML based on co‐mutation patterns to inform personalized treatment strategies and optimize outcomes.

These findings highlight potential synergistic effects between *ASXL1* and *KRAS* mutations. *ASXL1* mutations disrupt chromatin modification and transcriptional regulation. Chou et al. found that *ASXL1* mutations cluster with specific patient demographics and cytogenetic abnormalities, such as old age, secondary AML, and elevated peripheral blood leukocyte counts [2]. Moreover, these mutations frequently coexist with trisomy 8 and abnormalities of chromosome 11 while being negatively associated with myelodysplasia‐related cytogenetic abnormalities, particularly monosomy 5/del(5q) and monosomy 7/del(7q) [16]. Another study determined the genomic characteristics and prognostic significance of co‐mutated *ASXL1/SRSF2* AML and found that 60% of co‐mutated *ASXL1/SRSF2* cases were secondary cases, and the presence of these mutations was associated with a 1.4 and 1.6 times higher risk of death than that of patients with isolated *ASXL1* or *SRSF2* mutations, respectively [17]. This suggests that co‐mutated *ASXL1/SRSF2* AML represents a unique AML subgroup with distinct pathogenic mechanisms and leukemogenic processes compared to AML with single mutations. *KRAS* mutations drive oncogenic signaling. 11q23/KMT2A‐rearranged AML with *KRAS* mutation should be classified as high‐risk owing to the independent prognostic influence of *KRAS* mutations on the clinical outcomes of chemotherapy and HSCT treatment [18, 19]. Conversely, *RAS* mutations, which account for one‐third of CBF‐AML cases, have no impact on the OS of patients with *RAS*‐mutated CBF‐AML [20]. Together, these findings suggest that *ASXL1* or *KRAS* mutations co‐occurring with other gene mutations or cytogenetic abnormalities may or may not define distinct subgroups. However, *ASXL1* ^mut^/*KRAS* ^mut^ co‐mutations in AML as a unique molecular subgroup remain largely unexplored. These observations suggest that the molecular mechanisms underlying this co‐mutation warrant further investigation.

The mechanisms by which *ASXL1* and *KRAS* co‐mutations lead to worse outcomes are not fully understood, but we hypothesize a potential synergistic effect. *ASXL1* mutations are known to cause epigenetic dysregulation by affecting histone methylation, while *KRAS* mutations constitutively activate downstream signaling pathways, such as the *MAPK* and *PI3K/AKT* cascades, driving cell proliferation and survival. It is plausible that the epigenetic priming caused by *ASXL1* mutation, combined with the pro‐oncogenic signals from mutated *KRAS*, creates a more aggressive leukemic phenotype that is resistant to current therapies. Further research into the epigenetic and transcriptomic interplay between these two mutations is warranted to uncover the specific molecular pathways driving this synergy.

Notably, this study initially sought to provide evidence regarding the potential benefit of HSCT in improving the poor prognosis associated with *ASXL1* ^*mut*^ */KRAS* ^*mut*^ co‐mutated AML. However, a critical finding of our updated analysis is the significant impact of immortal time bias on these results. When a 3‐month landmark approach was applied to account for this bias, the survival benefit previously observed in patients who underwent HSCT was no longer evident. This shift in significance suggests that in clinical practice, the perceived survival advantage of HSCT in retrospective cohorts often includes patients who were inherently fit enough or survived long enough to receive the transplant. Furthermore, the limited sample size of our *ASXL1* ^*mut*^ */KRAS* ^*mut*^ subgroup restricts the statistical power to detect smaller therapeutic differences. Given the lack of statistical significance in the landmark model, our data suggest that HSCT may not provide a robust survival advantage for this specific high‐risk population. These findings underscore the urgent need for larger, prospective studies to clarify the true role of transplantation in overcoming the adverse impact of these co‐mutations.

This study has certain limitations. First, the *ASXL1* ^*mut*^ */KRAS* ^*mut*^ cohort size of 27 patients and the limited number of clinical events in this subgroup inherently restrict the statistical power and generalizability of our findings; this study represents the first comprehensive analysis of this rare molecular subset. The results, while preliminary, serve as a critical hypothesis‐generating finding that warrants validation in larger, multi‐center prospective studies. Second, the study relied on retrospective data, which may introduce selection bias. Prospective studies with larger cohorts are needed to confirm the observed prognostic impact of *ASXL1* ^*mut*^ */KRAS* ^*mut*^ and to refine risk stratification in AML. Furthermore, the mechanisms underlying the interaction between *ASXL1* and *KRAS* mutations remain incompletely understood, highlighting the need for functional studies to elucidate their role in leukemogenesis.

This study highlights the critical prognostic significance of co‐mutations in *ASXL1* and *KRAS* in patients with AML. Our findings demonstrate that the *ASXL1* ^mut^/*KRAS* ^mut^ co‐mutational profile defines a unique molecular subgroup characterized by significantly lower CR rates, higher relapse rates, shorter RFS, and shorter OS compared to cases with single *ASXL1* or *KRAS* mutations. Notably, HSCT emerged as a promising therapeutic intervention, mitigating the adverse outcomes associated with this aggressive molecular phenotype and extending survival. The survival benefit observed with HSCT underscores the importance of incorporating genomic profiling into clinical decision‐making to identify high‐risk patients and guide personalized treatment strategies. Future research should focus on unraveling the biological interplay between *ASXL1* and *KRAS* mutations and developing targeted therapies to improve outcomes for this challenging patient population.

## Author Contributions

**Yijing Zhao:** funding acquisition (lead), investigation (equal), methodology (equal), software (lead), validation (lead), writing – original draft (lead), writing – review and editing (lead). **Ting Zhao:** data curation (equal). **Feifei Tang:** data curation (equal), resources (equal). **Xiaodong Mo:** data curation (equal), resources (equal). **Yingjun Chang:** data curation (equal), resources (equal). **Xiaosu Zhao:** data curation (equal), resources (equal). **Lanping Xu:** data curation (equal), resources (equal). **Yu Wang:** data curation (equal), resources (equal). **Xiaohui Zhang:** data curation (equal), resources (equal). **Hao Jiang:** data curation (equal), resources (equal). **Qian Jiang:** data curation (equal), resources (equal). **Xiaojun Huang:** conceptualization (equal), investigation (equal), supervision (equal). **Yuqian Sun:** conceptualization (equal), investigation (lead), project administration (equal), resources (equal), supervision (equal), writing – original draft (equal), writing – review and editing (equal).

## Funding

This work was supported by the Beijing Nova Program Interdisciplinary Cooperation Project (0107050); the Peking University Medicine Fund of Fostering Young Scholars' Scientific & Technological Innovation; the Fundamental Research Funds for the Central Universities (BMU2021PYB005); the. Peking University People's Hospital Research and Development Funds (RDY2020‐29, RS2020‐03); the Beijing Nova Program of Science and Technology (Z211100002121058), and the National Natural Science Foundation of China (82100168).

## Ethics Statement

This study was approved by the ethics review board of Peking University People's Hospital and was conducted in accordance with the Declaration of Helsinki.

## Consent

All the patients signed the appropriate written consent.

## Conflicts of Interest

The authors declare no conflicts of interest.

## Supporting information


**Supplementary Figure 1** Prognostic comparison between *ASXL1* ^*mut*^ */KRAS* ^*mut*^ AML and other ELN‐2022 adverse‐risk subgroups. (A, B) Comparison of OS (*p* = 0.474) and RFS (*p* = 0.979) between the *ASXL1* ^*mut*^ */KRAS* ^*mut*^ group and patients with a complex karyotype. (C, D) Comparison of OS (*p* = 0.497) and RFS (*p* = 0.198) between the *ASXL1* ^*mut*^ */KRAS* ^*mut*^ and patients with *TP53*mutations. *p*‐values were calculated using the log‐rank test. Numbers at risk are indicated below each plot. Supplemental Table 1 List of deep‐targeted sequencing panel: From 175 genes (2018–2020) to 290 genes (2021 onwards). Supplemental Table 2 Genomic characteristics of co‐mutated *ASXL1/KRAS* AML. Supplemental Table 3 Pairwise comparisons of subgroups of *ASXL1* ^mut^/*KRAS* ^mut^ co‐mutation and single mutation for OS in the PKUPH datasets. Supplemental Table 4 Pairwise comparisons of subgroups of *ASXL1* ^*mut*^ */KRAS* ^*mut*^ co‐mutation reveal significant heterogeneity in the adverse subgroup for OS. Supplemental Table 5 Pairwise comparisons of subgroups of *ASXL1* ^mut^/*KRAS* ^mut^ co‐mutation and single mutation for RFS in the PKUPH datasets. Supplemental Table 6 Pairwise comparisons of subgroups of *ASXL1* ^mut^/*KRAS* ^mut^ co‐mutation and single mutation for OS in the Beat AML datasets.

## Data Availability

The data used in this study were obtained from the Beat AML project (https://www.vizome.org). The datasets analyzed during the current study are available from the corresponding author upon reasonable request.

## References

[1] K. Sasaki , R. Kanagal‐Shamanna , G. Montalban‐Bravo , et al., “Impact of the Variant Allele Frequency of ASXL1, DNMT3A, JAK2, TET2, TP53, and NPM1 on the Outcomes of Patients With Newly Diagnosed Acute Myeloid Leukemia,” Cancer 126, no. 4 (2020): 765–774. 31742675 10.1002/cncr.32566

[2] W. C. Chou , H. H. Huang , H. A. Hou , et al., “Distinct Clinical and Biological Features of de Novo Acute Myeloid Leukemia With Additional Sex Comb‐Like 1 (ASXL1) Mutations,” Blood 116, no. 20 (2010): 4086–4094, 10.1182/blood-2010-05-283291. 20693432

[3] J. W. Tyner , C. E. Tognon , D. Bottomly , et al., “Functional Genomic Landscape of Acute Myeloid Leukaemia,” Nature 562, no. 7728 (2018): 526–531, 10.1038/s41586-018-0623-z. 30333627 PMC6280667

[4] H. Dohner , A. H. Wei , F. R. Appelbaum , et al., “Diagnosis and Management of AML in Adults: 2022 Recommendations From an International Expert Panel on Behalf of the ELN,” Blood 140, no. 12 (2022): 1345–1377, 10.1182/blood.2022016867. 35797463

[5] Y. L. Zhou , L. X. Wu , R. Peter Gale , et al., “Mutation Topography and Risk Stratification for de Novo Acute Myeloid Leukaemia With Normal Cytogenetics and no Nucleophosmin 1 (NPM1) Mutation or Fms‐Like Tyrosine Kinase 3 Internal Tandem Duplication (FLT3‐ITD),” British Journal of Haematology 190, no. 2 (2020): 274–283, 10.1111/bjh.16526. 32103499

[6] S. Fan , Y. Yang , S. Lu , et al., “DEK‐NUP214 Monitoring Before and After Allogeneic Haematopoietic Stem Cell Transplantation for Acute Myeloid Leukemia: A Report From the TROPHY Study Group,” Journal of Translational Internal Medicine 13, no. 4 (2025): 375–385, 10.1515/jtim-2025-0032. 40861068 PMC12371398

[7] L. X. Wu , H. Jiang , Y. J. Chang , et al., “Risk Stratification of Cytogenetically Normal Acute Myeloid Leukemia With Biallelic CEBPA Mutations Based on a Multi‐Gene Panel and Nomogram Model,” Frontiers in Oncology 11 (2021): 706935, 10.3389/fonc.2021.706935. 34485141 PMC8415912

[8] W. J. Yu , J. S. Jia , J. Wang , et al., “Short‐Term Efficacy of Venetoclax Combined With Azacitidine in Acute Myeloid Leukemia: A Single‐Institution Experience,” Zhonghua Xue Ye Xue Za Zhi = Zhonghua Xueyexue Zazhi 43, no. 2 (2022): 134–140, 10.3760/cma.j.issn.0253-2727.2022.02.008. 35381674 PMC8980640

[9] W. Duan , X. Liu , X. Zhao , et al., “Both the Subtypes of KIT Mutation and Minimal Residual Disease Are Associated With Prognosis in Core Binding Factor Acute Myeloid Leukemia: A Retrospective Clinical Cohort Study in Single Center,” Annals of Hematology 100, no. 5 (2021): 1203–1212, 10.1007/s00277-021-04432-z. 33474629

[10] Y. Z. Qin , L. P. Xu , H. Chen , et al., “Allogeneic Stem Cell Transplant May Improve the Outcome of Adult Patients With Inv(16) Acute Myeloid Leukemia in First Complete Remission With Poor Molecular Responses to Chemotherapy,” Leukemia & Lymphoma 56, no. 11 (2015): 3116–3123, 10.3109/10428194.2015.1032964. 25804769

[11] H. H. Zhu , X. H. Zhang , Y. Z. Qin , et al., “MRD‐Directed Risk Stratification Treatment May Improve Outcomes of t(8;21) AML in the First Complete Remission: Results From the AML05 Multicenter Trial,” Blood 121, no. 20 (2013): 4056–4062, 10.1182/blood-2012-11-468348. 23535063

[12] L. Xu , H. Chen , J. Chen , et al., “The Consensus on Indications, Conditioning Regimen, and Donor Selection of Allogeneic Hematopoietic Cell Transplantation for Hematological Diseases in China‐Recommendations From the Chinese Society of Hematology,” Journal of Hematology & Oncology 11, no. 1 (2018): 33, 10.1186/s13045-018-0564-x. 29495966 PMC5833104

[13] W. J. Yu , Y. Q. Sun , L. P. Xu , et al., “Comparison of Outcomes for Patients With Acute Myeloid Leukemia Undergoing Haploidentical Stem Cell Transplantation in First and Second Complete Remission,” Annals of Hematology 102 (2023): 2241–2250, 10.1007/s00277-023-05324-0. 37344697

[14] W. X. Huo , Q. Wen , X. H. Zhang , et al., “Outcomes of Haploidentical Haematopoietic Stem Cell Transplantation for Adolescent and Young Adults With Acute Myeloid Leukaemia,” British Journal of Haematology 202 (2023): 856–865, 10.1111/bjh.18937. 37365147

[15] Y. Mu , X. Fan , T. Chen , et al., “MYD88‐Mutated Chronic Lymphocytic Leukaemia/Small Lymphocytic Lymphoma as a Distinctive Molecular Subgroup Is Associated With Atypical Immunophenotypes in Chinese Patients,” Journal of Clinical Medicine 12, no. 7 (2023): 2667, 10.3390/jcm12072667. 37048750 PMC10094974

[16] K. Kakosaiou , F. Panitsas , A. Daraki , et al., “ASXL1 Mutations in AML Are Associated With Specific Clinical and Cytogenetic Characteristics,” Leukemia & Lymphoma 59, no. 10 (2018): 2439–2446, 10.1080/10428194.2018.1433298. 29411666

[17] D. R. Richardson , D. M. Swoboda , D. T. Moore , et al., “Genomic Characteristics and Prognostic Significance of Co‐Mutated ASXL1/SRSF2 Acute Myeloid Leukemia,” American Journal of Hematology 96, no. 4 (2021): 462–470, 10.1002/ajh.26110. 33502020 PMC10284351

[18] S. Iyoda , K. Yoshida , K. Shoji , et al., “KRAS G12 Mutations as Adverse Prognostic Factors in KMT2A‐Rearranged Acute Myeloid Leukemia,” Leukemia 38, no. 7 (2024): 1609–1612, 10.1038/s41375-024-02244-4. 38632314

[19] Y. Wu , X. Yuan , X. Lai , et al., “KRAS Mutation Status Critically Determines the Clinical Outcome of Patients With KMT2A‐Rearranged Acute Myeloid Leukemia,” Cancer 131, no. 13 (2025): e35941, 10.1002/cncr.35941. 40530668

[20] H. Awada , C. Gurnari , M. Meggendorfer , et al., “AML‐156: The Clinical Implications and Outcomes of RAS Mutatome in Core‐Binding Factor Acute Myeloid Leukemia,” Clinical Lymphoma Myeloma and Leukemia 20 (2020): S186.

