# Supplementary material for: Co‐Mutation of ASXL1 and KRAS Defines a Novel Ultra‐Adverse‐Risk Subtype of Acute Myeloid Leukemia in a Large‐Scale Cohort
Source: Cancer Med. 2026 Mar 11;15(3):e71715. doi: 10.1002/cam4.71715 (PMC13093774; doi:10.1002/cam4.71715)
Supplement: Supplementary file 1 — Supplementary Figure 1 Prognostic comparison between ASXL1 mut /KRAS mut AML and other ELN‐2022 adverse‐risk subgroups. (A, B) Comparison of OS (p = 0.474) and RFS (p = 0.979) between the ASXL1 mut /KRAS mut group and patients with a complex karyotype. (C, D) Comparison of OS (p = 0.497) and RFS (p = 0.198) between the ASXL1 mut /KRAS mut and patients with TP53mutations. p‐values were calculated using the log‐rank test. Numbers at risk are indicated below each plot. Supplemental Table 1 List of deep‐targeted sequencing panel: From 175 genes (2018–2020) to 290 genes (2021 onwards). Supplemental Table 2 Genomic characteristics of co‐mutated ASXL1/KRAS AML. Supplemental Table 3 Pairwise comparisons of subgroups of ASXL1 mut/KRAS mut co‐mutation and single mutation for OS in the PKUPH datasets. Supplemental Table 4 Pairwise comparisons of subgroups of ASXL1 mut /KRAS mut co‐mutation reveal significant heterogeneity in the adverse subgroup for OS. Supplemental Table 5 Pairwise comparisons of subgroups of ASXL1 mut/KRAS mut co‐mutation and single mutation for RFS in the PKUPH datasets. Supplemental Table 6 Pairwise comparisons of subgroups of ASXL1 mut/KRAS mut co‐mutation and single mutation for OS in the Beat AML datasets. [file CAM4-15-e71715-s001.docx]

**Supplementary Figure**

**
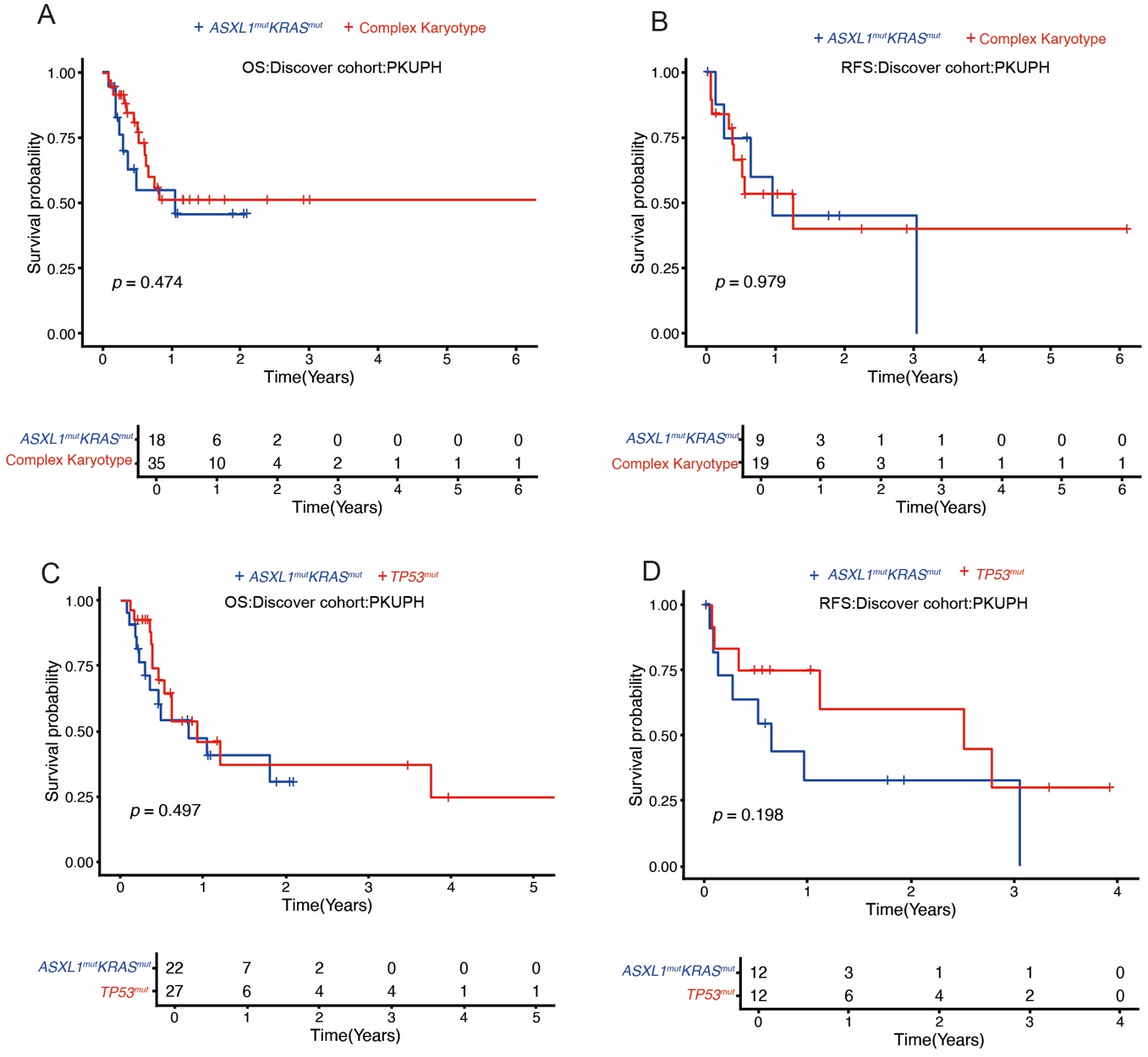
**

**Supplementary Figure 1. Prognostic comparison between *ASXL1^mut^/KRAS^mut^* AML and other ELN-2022 adverse-risk subgroups.**

(A-B) Comparison of OS ( *p* = 0.474) and RFS ( *p* = 0.979) between the ***ASXL1^mut^/KRAS^mut^*** group and patients with a complex karyotype.

(C-D) Comparison of OS ( *p* = 0.497) and RFS ( *p* = 0.198) between the ***ASXL1^mut^/KRAS^mut^*** and patients with *TP53*mutations. *p* values were calculated using the log-rank test. Numbers at risk are indicated below each plot.

**Supplementary Tables**

**Supplemental Table 1. List of deep-targeted sequencing panel: from 175 genes (2018–2020) to 290 genes (2021 onwards)**

| **175 genes list (2018–2020)** | | | | | | | | | **Expanded to 290 genes list (2021 onwards)** | | | | | |
| --- | --- | --- | --- | --- | --- | --- | --- | --- | --- | --- | --- | --- | --- | --- |
| *ABCB1* | *BRAF* | *CEBPA* | *EPHA7* | *HRAS* | *KRAS* | *PAX5* | *SBDS* | *U2AF1* | *AKT3* | *CHD2* | *GATA3* | *MSH2* | *PRKD2* | *TYK2* |
| *ABL1* | *BRCA1* | *CHD8* | *EPOR* | *ID3* | *KRT20* | *PDGFRA* | *SETBP1* | *VHL* | *ALK* | *CHEK2* | *HFE* | *MSH6* | *PTCH1* | *U2AF2* |
| *ANKRD26* | *BRCA2* | *CIITA* | *ETV6* | *IDH1* | *LMO2* | *PDGFRB* | *SETD2* | *WHSC1* | *ARID5B* | *CIC* | *HIST1H1B* | *NBN* | *PTPN2* | *UBR5* |
| *APC* | *BRIP1* | *CREBBP* | *EZH2* | *IDH2* | *LYN* | *PHF6* | *SETDB1* | *WT1* | *ASXL2* | *CYLD* | *HIST1H1C* | *NFKBIA* | *PTPN6* | *WAS* |
| *ARID1A* | *BTG1* | *CRLF2* | *FAM46C* | *IKZF1* | *MAP2K1* | *PIGA* | *SF3B1* | *XPO1* | *ATP6V1B2* | *DDX3X* | *HIST1H1D* | *NFKBIE* | *PTPRD* | *ZFHX4* |
| *ARID1B* | *BTK* | *CSF1R* | *FAS* | *IKZF2* | *MCL1* | *PIK3CA* | *SGK1* | *ZAP70* | *AXIN1* | *DHFR* | *HIST1H1E* | *NOTCH3* | *PTPRT* | *ZMYM3* |
| *ARID2* | *CALR* | *CSF3R* | *FAT1* | *IKZF3* | *MEF2B* | *PIK3CD* | *SH2B3* | *ZRSR2* | *BAX* | *DNAH10* | *HUWE1* | *NOTCH4* | *RAD50* | *SPEN* |
| *ASXL1* | *CARD11* | *CTCF* | *FBXO11* | *IL7R* | *MFHAS1* | *PIM1* | *SMC1A* |  | *BCL11B* | *DOT1L* | *IGLL5* | *NTRK1* | *RARA* | *TBL1XR1* |
| *ATG2B* | *CBL* | *CUX1* | *FBXW7* | *IRF4* | *MPL* | *PLCG2* | *SMC3* |  | *BCL7A* | *DTX1* | *IKBKB* | *NTRK2* | *RB1* | *TET1* |
| *ATM* | *CBLB* | *CXCR4* | *FLT3* | *IRF8* | *MTOR* | *PPM1D* | *SOCS1* |  | *BLNK* | *DUSP2* | *ITK* | *NTRK3* | *RPL10* | *TMEM30A* |
| *ATRX* | *CBLC* | *DDX41* | *FOXO1* | *ITPKB* | *MYC* | *PRDM1* | *SRP72* |  | *BRD4* | *EBF1* | *JUNB* | *NUDT15* | *RPS15* | *TRAF5* |
| *B2M* | *CCND1* | *DIS3* | *GATA1* | *JAK1* | *MYD88* | *PRF1* | *SRSF2* |  | *BTG2* | *EGR1* | *KAT6A* | *P2RY8* | *RRAGC* |  |
| *BCL10* | *CCND3* | *DKC1* | *GATA2* | *JAK2* | *MYOM2* | *PRKDC* | *STAG2* |  | *CACNA1H* | *EGR2* | *KLF2* | *PIK3R1* | *RTEL1* |  |
| *BCL2* | *CD28* | *DNM2* | *GFI1* | *JAK3* | *NF1* | *PRPF8* | *STAT3* |  | *CCR4* | *ERBB3* | *KLHL6* | *PIM2* | *SETD1B* |  |
| *BCL6* | *CD58* | *DNMT3A* | *GNA13* | *KDM6A* | *NOTCH1* | *PTEN* | *STAT5B* |  | *CD22* | *ERG* | *LTB* | *PLCG1* | *SF1* |  |
| *BCOR* | *CD79A* | *EED* | *GNAI2* | *KIT* | *NOTCH2* | *PTPN11* | *STAT6* |  | *CD70* | *ETNK1* | *MAX* | *PML* | *SF3A1* |  |
| *BCORL1* | *CD79B* | *EGFR* | *GNAS* | *KMT2A* | *NPM1* | *RAD21* | *SUZ12* |  | *CDC25C* | *FAT3* | *MDM2* | *PMS2* | *SH2D1A* |  |
| *BIRC3* | *CDKN1A* | *EGLN1* | *GNB1* | *KMT2B* | *NRAS* | *RELN* | *SYK* |  | *CDK6* | *FAT4* | *MED12* | *POT1* | *SLC29A1* |  |
| *BLM* | *CDKN2A* | *ELANE* | *GSKIP* | *KMT2C* | *NT5C2* | *RHOA* | *TAL1* |  | *CDKN1B* | *FGFR3* | *MGA* | *POU2AF1* | *SMARCA4* |  |
| *BPGM* | *CDKN2B* | *EP300* | *HAX1* | *KMT2D* | *PALB2* | *RUNX1* | *TCF3* |  | *CEBPE* | *G6PC3* | *MLH1* | *PRKCB* | *SMARCB1* |  |
| *TERT* | *TERC* | *TET2* | *TNFAIP3* | *TNFRSF14* | *TP53* | *TPMT* | *TRAF3* |  | *SP140* | *STAT5A* | *TCF3* | *TINF2* | *TRAF2* |  |

**Supplemental Table 2.Genomic characteristics characters of co-mutated *ASXL1/KRAS* AML**

| Number | Level | Overall | *ASXL1^mut^KRAS^wt^* | *ASXL1^mut^KRAS^mut^* | *ASXL1^wt^KRAS^mut^* | p-value |
| --- | --- | --- | --- | --- | --- | --- |
| N |  | **394** | **201** | **22** | **171** |  |
| *CEBPA-bZIP (%)* | **WT** | 383 (97.2) | 194 (96.5) | 22 (100.0) | 167 (97.7) | 0.57 |
|  | **Mutation** | 11 (2.8) | 7 (3.5) | 0 (0.0) | 4 (2.3) |  |
| *TP53 (%)* | **WT** | 367 (93.1) | 182 (90.5) | 22 (100.0) | 163 (95.3) | 0.08 |
|  | **Mutation** | 27 (6.9) | 19 (9.5) | 0 (0.0) | 8 (4.7) |  |
| *NPM1 (%)* | **WT** | 363 (92.1) | 195 (97.0) | 21 (95.5) | 147 (86.0) | <0.01 |
|  | **Mutation** | 31 (7.9) | 6 (3.0) | 1 (4.5) | 24 (14.0) |  |
| *FLT3-ITD (%)* | **WT** | 362 (91.9) | 190 (94.5) | 21 (95.5) | 151 (88.3) | 0.07 |
|  | **Mutation** | 32 (8.1) | 11 (5.5) | 1 (4.5) | 20 (11.7) |  |
| *FLT3-TKD (%)* | **WT** | 340 (91.2) | 183 (95.3) | 19 (90.5) | 138 (86.2) | 0.01 |
|  | **Mutation** | 33 (8.8) | 9 (4.7) | 2 (9.5) | 22 (13.8) |  |
| *ASXL1 (%)* | **WT** | 171 (43.4) | 0 (0.0) | 0 (0.0) | 171 (100.0) | <0.01 |
|  | **Mutation** | 223 (56.6) | 201 (100.0) | 22 (100.0) | 0 (0.0) |  |
| *KRAS (%)* | **WT** | 201 (51.0) | 201 (100.0) | 0 (0.0) | 0 (0.0) | <0.01 |
|  | **Mutation** | 193 (49.0) | 0 (0.0) | 22 (100.0) | 171 (100.0) |  |
| *BCOR (%)* | **WT** | 365 (92.6) | 189 (94.0) | 18 (81.8) | 158 (92.4) | 0.11 |
|  | **Mutation** | 29 (7.4) | 12 (6.0) | 4 (18.2) | 13 (7.6) |  |
| *SF3B1 (%)* | **WT** | 389 (98.7) | 199 (99.0) | 22 (100.0) | 168 (98.2) | 0.7 |
|  | **Mutation** | 5 (1.3) | 2 (1.0) | 0 (0.0) | 3 (1.8) |  |
| *U2AF1 (%)* | **WT** | 347 (88.1) | 170 (84.6) | 13 (59.1) | 164 (95.9) | <0.01 |
|  | **Mutation** | 47 (11.9) | 31 (15.4) | 9 (40.9) | 7 (4.1) |  |
| *STAG2 (%)* | **WT** | 373 (94.7) | 181 (90.0) | 22 (100.0) | 170 (99.4) | <0.01 |
|  | **Mutation** | 21 (5.3) | 20 (10.0) | 0 (0.0) | 1 (0.6) |  |
| *EZH2 (%)* | **WT** | 382 (97.0) | 191 (95.0) | 21 (95.5) | 170 (99.4) | 0.04 |
|  | **Mutation** | 12 (3.0) | 10 (5.0) | 1 (4.5) | 1 (0.6) |  |
| *SRSF2 (%)* | **WT** | 355 (90.1) | 164 (81.6) | 20 (90.9) | 171 (100.0) | <0.01 |
|  | **Mutation** | 39 (9.9) | 37 (18.4) | 2 (9.1) | 0 (0.0) |  |
| *RUNX1 (%)* | **WT** | 344 (87.3) | 162 (80.6) | 18 (81.8) | 164 (95.9) | <0.01 |
|  | **Mutation** | 50 (12.7) | 39 (19.4) | 4 (18.2) | 7 (4.1) |  |
| *ZRSR2 (%)* | **WT** | 390 (99.0) | 198 (98.5) | 21 (95.5) | 171 (100.0) | 0.08 |
|  | **Mutation** | 4 (1.0) | 3 (1.5) | 1 (4.5) | 0 (0.0) |  |
| *BCORL1 (%)* | **WT** | 382 (97.0) | 199 (99.0) | 22 (100.0) | 161 (94.2) | 0.02 |
|  | **Mutation** | 12 (3.0) | 2 (1.0) | 0 (0.0) | 10 (5.8) |  |
| *BRAF (%)* | **WT** | 389 (98.7) | 198 (98.5) | 21 (95.5) | 170 (99.4) | 0.27 |
|  | **Mutation** | 5 (1.3) | 3 (1.5) | 1 (4.5) | 1 (0.6) |  |
| *CBL (%)* | **WT** | 367 (93.1) | 188 (93.5) | 19 (86.4) | 160 (93.6) | 0.43 |
|  | **Mutation** | 27 (6.9) | 13 (6.5) | 3 (13.6) | 11 (6.4) |  |
| *CSF3R (%)* | **WT** | 375 (95.2) | 185 (92.0) | 22 (100.0) | 168 (98.2) | 0.01 |
|  | **Mutation** | 19 (4.8) | 16 (8.0) | 0 (0.0) | 3 (1.8) |  |
| *DNMT3A (%)* | **WT** | 350 (88.8) | 186 (92.5) | 21 (95.5) | 143 (83.6) | 0.01 |
|  | **Mutation** | 44 (11.2) | 15 (7.5) | 1 (4.5) | 28 (16.4) |  |
| *ETV6 (%)* | **WT** | 385 (97.7) | 195 (97.0) | 20 (90.9) | 170 (99.4) | 0.03 |
|  | **Mutation** | 9 (2.3) | 6 (3.0) | 2 (9.1) | 1 (0.6) |  |
| *GATA2 (%)* | **WT** | 377 (95.7) | 190 (94.5) | 21 (95.5) | 166 (97.1) | 0.48 |
|  | **Mutation** | 17 (4.3) | 11 (5.5) | 1 (4.5) | 5 (2.9) |  |
| *IDH2 (%)* | **WT** | 358 (90.9) | 179 (89.1) | 21 (95.5) | 158 (92.4) | 0.4 |
|  | **Mutation** | 36 (9.1) | 22 (10.9) | 1 (4.5) | 13 (7.6) |  |
| *KIT (%)* | **WT** | 349 (88.6) | 186 (92.5) | 22 (100.0) | 141 (82.5) | <0.01 |
|  | **Mutation** | 45 (11.4) | 15 (7.5) | 0 (0.0) | 30 (17.5) |  |
| *KMT2D (%)* | **WT** | 389 (98.7) | 200 (99.5) | 20 (90.9) | 169 (98.8) | <0.01 |
|  | **Mutation** | 5 (1.3) | 1 (0.5) | 2 (9.1) | 2 (1.2) |  |
| *NRAS (%)* | **WT** | 284 (72.1) | 171 (85.1) | 14 (63.6) | 99 (57.9) | <0.01 |
|  | **Mutation** | 110 (27.9) | 30 (14.9) | 8 (36.4) | 72 (42.1) |  |
| *PHF6 (%)* | **WT** | 374 (94.9) | 186 (92.5) | 20 (90.9) | 168 (98.2) | 0.03 |
|  | **Mutation** | 20 (5.1) | 15 (7.5) | 2 (9.1) | 3 (1.8) |  |
| *SETBP1 (%)* | **WT** | 373 (94.7) | 182 (90.5) | 21 (95.5) | 170 (99.4) | <0.01 |
|  | **Mutation** | 21 (5.3) | 19 (9.5) | 1 (4.5) | 1 (0.6) |  |
| *SETD2 (%)* | **WT** | 386 (98.0) | 194 (96.5) | 22 (100.0) | 170 (99.4) | 0.11 |
|  | **Mutation** | 8 (2.0) | 7 (3.5) | 0 (0.0) | 1 (0.6) |  |
| *TET2 (%)* | **WT** | 335 (85.0) | 155 (77.1) | 18 (81.8) | 162 (94.7) | <0.01 |
|  | **Mutation** | 59 (15.0) | 46 (22.9) | 4 (18.2) | 9 (5.3) |  |
| *PTPN11 (%)* | **WT** | 358 (90.9) | 195 (97.0) | 16 (72.7) | 147 (86.0) | <0.01 |
|  | **Mutation** | 36 (9.1) | 6 (3.0) | 6 (27.3) | 24 (14.0) |  |
| *JAK2 (%)* | **WT** | 380 (96.4) | 190 (94.5) | 21 (95.5) | 169 (98.8) | 0.08 |
|  | **Mutation** | 14 (3.6) | 11 (5.5) | 1 (4.5) | 2 (1.2) |  |
| MK (%) | **No** | 365 (92.6) | 182 (90.5) | 18 (81.8) | 165 (96.5) | 0.01 |
|  | **Yes** | 29 (7.4) | 19 (9.5) | 4 (18.2) | 6 (3.5) |  |
| t(v;11q23) (%) | **No** | 379 (96.2) | 196 (97.5) | 20 (90.9) | 163 (95.3) | 0.22 |
|  | **Yes** | 15 (3.8) | 5 (2.5) | 2 (9.1) | 8 (4.7) |  |
| t(9;11) (%) | **No** | 378 (95.9) | 197 (98.0) | 21 (95.5) | 160 (93.6) | 0.1 |
|  | **Yes** | 16 (4.1) | 4 (2.0) | 1 (4.5) | 11 (6.4) |  |
| t(3q26;v) (%) | **No** | 390 (99.0) | 200 (99.5) | 21 (95.5) | 169 (98.8) | 0.19 |
|  | **Yes** | 4 (1.0) | 1 (0.5) | 1 (4.5) | 2 (1.2) |  |
| inv (16)/t(16;16) (%) | **No** | 346 (87.8) | 199 (99.0) | 22 (100.0) | 125 (73.1) | <0.01 |
|  | **Yes** | 48 (12.2) | 2 (1.0) | 0 (0.0) | 46 (26.9) |  |
| t(8;21) (%) | **No** | 349 (88.6) | 167 (83.1) | 22 (100.0) | 160 (93.6) | <0.01 |
|  | **Yes** | 45 (11.4) | 34 (16.9) | 0 (0.0) | 11 (6.4) |  |
| inv (3)/t(3;3) (%) | **No** | 385 (97.7) | 198 (98.5) | 19 (86.4) | 168 (98.2) | <0.01 |
|  | **Yes** | 9 (2.3) | 3 (1.5) | 3 (13.6) | 3 (1.8) |  |
| Complex karyotype (%) | **No** | 355 (90.1) | 177 (88.1) | 18 (81.8) | 160 (93.6) | 0.08 |
|  | **Yes** | 39 (9.9) | 24 (11.9) | 4 (18.2) | 11 (6.4) |  |
| -5/del(5q)/t(5q)/  add(5q) (%) | **No** | 381 (96.7) | 195 (97.0) | 21 (95.5) | 165 (96.5) | 0.91 |
|  | **Yes** | 13 (3.3) | 6 (3.0) | 1 (4.5) | 6 (3.5) |  |
| -7/del(7q) (%) | **No** | 367 (93.1) | 188 (93.5) | 17 (77.3) | 162 (94.7) | 0.01 |
|  | **Yes** | 27 (6.9) | 13 (6.5) | 5 (22.7) | 9 (5.3) |  |
| Trisomy 8 (%) | **No** | 354 (89.8) | 175 (87.1) | 17 (77.3) | 162 (94.7) | 0.01 |
|  | **Yes** | 40 (10.2) | 26 (12.9) | 5 (22.7) | 9 (5.3) |  |
| del(12p)/t(12p)/add(12p) (%) | **No** | 383 (97.2) | 195 (97.0) | 22 (100.0) | 166 (97.1) | 0.72 |
|  | **Yes** | 11 (2.8) | 6 (3.0) | 0 (0.0) | 5 (2.9) |  |
| –17/add(17p)/  del(17p) (%) | **No** | 380 (96.4) | 193 (96.0) | 22 (100.0) | 165 (96.5) | 0.63 |
|  | **Yes** | 14 (3.6) | 8 (4.0) | 0 (0.0) | 6 (3.5) |  |
| i17q (%) | **No** | 388 (98.5) | 195 (97.0) | 22 (100.0) | 171 (100.0) | 0.05 |
|  | **Yes** | 6 (1.5) | 6 (3.0) | 0 (0.0) | 0 (0.0) |  |
| del(20q) (%) | **No** | 386 (98.0) | 195 (97.0) | 21 (95.5) | 170 (99.4) | 0.18 |
|  | **Yes** | 8 (2.0) | 6 (3.0) | 1 (4.5) | 1 (0.6) |  |
| ELN2022 (%) | | | | | | |
| Favorable | | 124 (31.5) | 45 (22.4) | 0 (0.0) | 79 (46.2) | <0.01 |
| Intermediate | | 39 (9.9) | 3 (1.5) | 0 (0.0) | 36 (21.1) |  |
| Adverse | | 231 (58.6) | 153 (76.1) | 22 (100.0) | 56 (32.7) |  |

**Supplemental Table 3. Pairwise comparisons of subgroups of *ASXL1*^mut^/*KRAS*^mut^ co-mutation and single mutation for OS in the PKUPH datasets**

| **Pairwise Comparisons** | | | | | | | |
| --- | --- | --- | --- | --- | --- | --- | --- |
|  | ***ASXL1*^mut^/*KRAS*^mut^ in OS** | ***ASXL1*^mut^/*KRAS*^wt^** | | ***ASXL1*^mut^/*KRAS*^mut^** | | ***ASXL1*^wt^/*KRAS*^mut^** | |
|  |  | Chi-Square | p-value | Chi-Square | p-value | Chi-Square | p-value |
| Log Rank (Mantel–Cox) | ***ASXL1*^mut^/*KRAS*^wt^** |  |  | 11.477 | <0.001 | 8.355 | 0.004 |
|  | ***ASXL1*^mut^/*KRAS*^mut^** | 11.477 | <0.001 |  |  | 25.503 | <0.001 |
|  | ***ASXL1*^wt^/*KRAS*^mut^** | 8.355 | 0.004 | 25.503 | <0.001 |  |  |
| Breslow (Generalized Wilcoxon) | ***ASXL1*^mut^/*KRAS*^wt^** |  |  | 14.189 | <0.001 | 6.925 | 0.009 |
|  | ***ASXL1*^mut^/*KRAS*^mut^** | 14.189 | <0.001 |  |  | 29.375 | <0.001 |
|  | ***ASXL1*^wt^/*KRAS*^mut^** | 6.925 | 0.009 | 29.375 | <0.001 |  |  |
| Tarone–Ware | ***ASXL1*^mut^/*KRAS*^wt^** |  |  | 13.057 | <0.001 | 7.614 | 0.006 |
|  | ***ASXL1*^mut^/*KRAS*^mut^** | 13.057 | <0.001 |  |  | 27.974 | <0.001 |
|  | ***ASXL1*^wt^/*KRAS*^mut^** | 7.614 | 0.006 | 27.974 | <0.001 |  |  |

OS, overall survival

**Supplemental Table 4. Pairwise comparisons of subgroups of *ASXL1^mut^/KRAS^mut^* co-mutation reveal significant heterogeneity in adverse subgroup for OS**

| **Pairwise Comparisons** | | | | | | | | | |
| --- | --- | --- | --- | --- | --- | --- | --- | --- | --- |
|  | ***ASXL1*^mut^/*KRAS*^mut^ in ELN2022 for OS** | **FAV risk category** | | **INT risk category** | | **ADV risk category** | | ***ASXL1*^mut^/*KRAS*^mut^** | |
|  |  | Chi-Square | p-value | Chi-Square | p-value | Chi-Square | p-value | Chi-Square | p-value |
| Log Rank (Mantel–Cox) | **FAV** |  |  | 18.440 | <0.001 | 40.479 | <0.001 | 73.367 | <0.001 |
|  | **INT** | 18.440 | <0.001 |  |  | 0.409 | 0.523 | 6.072 | 0.014 |
|  | **ADV** | 40.479 | <0.001 | 0.409 | 0.523 |  |  | 10.107 | 0.001 |
|  | ***ASXL1*^mut^/*KRAS*^mut^** | 73.367 | <0.001 | 6.072 | 0.014 | 10.107 | 0.001 |  |  |
| Breslow (Generalized Wilcoxon) | **FAV** |  |  | 23.337 | <0.001 | 39.974 | <0.001 | 70.847 | <0.001 |
|  | **INT** | 23.337 | <0.001 |  |  | 0.060 | 0.807 | 5.465 | 0.019 |
|  | **ADV** | 39.974 | <0.001 | 0.060 | 0.807 |  |  | 11.969 | <0.001 |
|  | ***ASXL1*^mut^/*KRAS*^mut^** | 70.847 | <0.001 | 5.465 | 0.019 | 11.969 | <0.001 |  |  |
| Tarone–Ware | **FAV** |  |  | 21.606 | <0.001 | 42.156 | <0.001 | 72.890 | <0.001 |
|  | **INT** | 21.606 | <0.001 |  |  | 0.183 | 0.669 | 5.900 | 0.015 |
|  | **ADV** | 42.156 | <0.001 | 0.183 | 0.669 |  |  | 11.273 | <0.001 |
|  | ***ASXL1*^mut^/*KRAS*^mut^** | 72.890 | <0.001 | 5.900 | 0.015 | 11.273 | <0.001 |  |  |

FAV, Favorable; INT, Intermediate; ADV, Adverse.

**Supplemental Table 5. Pairwise comparisons of subgroups of *ASXL1*^mut^/*KRAS*^mut^ co-mutation and single mutation for RFS in the PKUPH datasets**

| **Pairwise Comparisons** | | | | | | | |
| --- | --- | --- | --- | --- | --- | --- | --- |
|  | ***ASXL1*^mut^/*KRAS*^mut^ in RFS** | ***ASXL1*^mut^/*KRAS*^wt^** | | ***ASXL1*^mut^/*KRAS*^mut^** | | ***ASXL1*^wt^/*KRAS*^mut^** | |
|  |  | Chi-Square | p-value | Chi-Square | p-value | Chi-Square | p-value |
| Log Rank (Mantel–Cox) | ***ASXL1*^mut^/*KRAS*^wt^** |  |  | 7.078 | 0.008 | 2.178 | 0.140 |
|  | ***ASXL1*^mut^/*KRAS*^mut^** | 7.078 | 0.008 |  |  | 12.714 | <0.001 |
|  | ***ASXL1*^wt^/*KRAS*^mut^** | 2.178 | 0.140 | 12.714 | <0.001 |  |  |
| Breslow (Generalized Wilcoxon) | ***ASXL1*^mut^/*KRAS*^wt^** |  |  | 7.264 | 0.007 | 1.138 | 0.286 |
|  | ***ASXL1*^mut^/*KRAS*^mut^** | 7.264 | 0.007 |  |  | 11.229 | <0.001 |
|  | ***ASXL1*^wt^/*KRAS*^mut^** | 1.138 | 0.286 | 11.229 | <0.001 |  |  |
| Tarone–Ware | ***ASXL1*^mut^/*KRAS*^wt^** |  |  | 7.174 | 0.007 | 1.583 | 0.208 |
|  | ***ASXL1*^mut^/*KRAS*^mut^** | 7.174 | 0.007 |  |  | 11.575 | <0.001 |
|  | ***ASXL1*^wt^/*KRAS*^mut^** | 1.583 | 0.208 | 11.575 | <0.001 |  |  |

RFS, relapse-free survival

**Supplemental Table 6. Pairwise comparisons of subgroups of *ASXL1*^mut^/*KRAS*^mut^ co-mutation and single mutation for OS in the Beat AML datasets**

| **Pairwise Comparisons** | | | | | | | |
| --- | --- | --- | --- | --- | --- | --- | --- |
|  | ***ASXL1*^mut^/*KRAS*^mut^ in OS** | ***ASXL1*^mut^/*KRAS*^mut^** | | ***ASXL1*^mut^/*KRAS*^wt^** | | ***ASXL1*^wt^/*KRAS*^mut^** | |
|  |  | Chi-Square | p-value | Chi-Square | p-value | Chi-Square | p-value |
| Log Rank (Mantel–Cox) | ***ASXL1*^mut^/*KRAS*^mut^** |  |  | 5.417 | 0.02 | 5.448 | 0.02 |
|  | ***ASXL1*^mut^/*KRAS*^wt^** | 5.417 | 0.02 |  |  | 0.834 | 0.361 |
|  | ***ASXL1*^wt^/*KRAS*^mut^** | 5.448 | 0.02 | 0.834 | 0.361 |  |  |
| Breslow (Generalized Wilcoxon) | ***ASXL1*^mut^/*KRAS*^mut^** |  |  | 3.98 | 0.046 | 3.557 | 0.059 |
|  | ***ASXL1*^mut^/*KRAS*^wt^** | 3.98 | 0.046 |  |  | 0.352 | 0.553 |
|  | ***ASXL1*^wt^/*KRAS*^mut^** | 3.557 | 0.059 | 0.352 | 0.553 |  |  |
| Tarone–Ware | ***ASXL1^mut^*/*KRAS*^mut^** |  |  | 4.627 | 0.031 | 4.404 | 0.036 |
|  | ***ASXL1*^mut^/*KRAS^wt^*** | 4.627 | 0.031 |  |  | 0.551 | 0.458 |
|  | ***ASXL1*^wt^/*KRAS*^mut^** | 4.404 | 0.036 | 0.551 | 0.458 |  |  |

OS, overall survival
